# Gene Expression in the Rodent Brain is Associated with Its Regional Connectivity

**DOI:** 10.1371/journal.pcbi.1002040

**Published:** 2011-05-05

**Authors:** Lior Wolf, Chen Goldberg, Nathan Manor, Roded Sharan, Eytan Ruppin

**Affiliations:** 1 Blavatnik School of Computer Science, Tel-Aviv University, Tel-Aviv, Israel; 2 School of Medicine, Tel-Aviv University, Tel-Aviv, Israel; Indiana University, United States of America

## Abstract

The putative link between gene expression of brain regions and their neural connectivity patterns is a fundamental question in neuroscience. Here this question is addressed in the first large scale study of a prototypical mammalian rodent brain, using a combination of rat brain regional connectivity data with gene expression of the mouse brain. Remarkably, even though this study uses data from two different rodent species (due to the data limitations), we still find that the connectivity of the majority of brain regions is highly predictable from their gene expression levels–the outgoing (incoming) connectivity is successfully predicted for 73% (56%) of brain regions, with an overall fairly marked accuracy level of 0.79 (0.83). Many genes are found to play a part in predicting both the incoming and outgoing connectivity (241 out of the 500 top selected genes, *p*-value<1e-5). Reassuringly, the genes previously known from the literature to be involved in axon guidance do carry significant information about regional brain connectivity. Surveying the genes known to be associated with the pathogenesis of several brain disorders, we find that those associated with schizophrenia, autism and attention deficit disorder are the most highly enriched in the connectivity-related genes identified here. Finally, we find that the profile of functional annotation groups that are associated with regional connectivity in the rodent is significantly correlated with the annotation profile of genes previously found to determine neural connectivity in *C. elegans* (Pearson correlation of 0.24, *p*<1e-6 for the outgoing connections and 0.27, *p*<1e-5 for the incoming). Overall, the association between connectivity and gene expression in a specific extant rodent species' brain is likely to be even stronger than found here, given the limitations of current data.

## Author Summary

Brain connectivity is believed to be associated with gene expression levels in the developing and the adult animal. Recently, this association has been explored in two model animals: the worm *C. elegans* at the level of single neurons; and the mouse, where specific subpopulations of neurons in the hippocampus were studied. Inspired by these studies, we set out to generalize their scope and examine the possibility of using gene expression signatures to predict regional connectivity in the whole rodent brain. Our results show a higher degree of association between connectivity and expression than shown before, and key genes are identified that are highly predictive of brain connectivity.

## Introduction

Genes play a major role in the formation of the nervous system and in its continuous function. They specify neuronal cell types, help destine neurons into defined neural circuits, and provide important cues determining their connectivity [1]–[2]. Inspired by Roger Sperry's classical chemo-affinity hypothesis that states that neuronal wiring takes place by selective attachment guided by specific molecular identifiers, a large array of studies have described various gene families that are involved in axonal guidance and in determining their specific targets (see [3]–[7] for reviews). Another central paradigm has posited that a central driving force in determining synaptic connectivity are activity-dependent mechanisms, by which synapses are formed between neurons whose firing tends to be correlated in a self-organizing Hebbian manner (see [8]–[9] for reviews). A third paradigm has recently emphasized the potential role of random axonal outgrowth and location-dependent competition in establishing connectivity [10]. These paradigms are obviously not mutually exclusive and are likely to concur concomitantly, and quantifying the extent of association between gene expression and connectivity may provide global constraints on their relative contribution.

A few recent studies have examined the association between gene expression and connectivity on the neuronal level in the worm *C. elegans*, by studying the relation between a neuron's gene expression and its connectivity to and from other neurons. *C. elegans* offers a unique opportunity to perform such an investigation, as it is currently the only model organism for which both a large fraction of its synaptic connectivity and gene expression are known on an individual neuronal level. While [11]–[12] have set to predict the formation of synapses in the worm based on the expression pattern of the pertaining genes [13], aimed to do so while additionally considering their spatial proximity. Overall, these studies have shown that: (1) neuronal gene expression does contain significant information about its connectivity, but the predictive power it entails is rather moderate, at least with the current available data, and (2) it is still possible to use this information to identify genes that potentially play part in determining the neural architecture, on a genome scale. Here we aim to significantly go beyond these earlier studies and to investigate the fundamental relation between gene expression and connectivity in a *mammalian* brain, and to study it at the level of connectivity between different brain regions.

A recent study [14] has used the mouse brain data of the Allen mouse brain atlas (ABA) [15]–[16] and the accompanying spatial gene expression correlation map tool to study gene expression patterns within the CA1 field. Multiple observations have been made to suggest that gene expression associations between CA1 regions and other sub-cortical brain regions are indicative of direct or indirect projections to or from distinct spatial domains of the CA1 field. In another study [17], it was shown that a factorization of the hippocampus volume by the local gene expression levels leads to a spatial grouping that agrees with the known patterns of differential connectivity. Inspired by these studies, we set out here to generalize their scope and examine the possibility of using gene expression signatures to predict regional connectivity in a mammalian brain. Presently, as there is no adequate regional gene expression and connectivity data available for a single mammalian species, we therefore fuse data from two species: brain wiring data for the rat brain and regional gene expression data from the mouse brain, to study their relation in a prototypical rodent brain. The rat connectivity atlas [18] available online (http://brancusi.usc.edu/bkms/) provides connectivity information for the anatomical structures of the rat. The Allen mouse brain atlas (ABA) [15]–[16] provides gene expression images for the adult mouse brain. Although gene expression during embryogenesis and development would have ideally been more befitting, this data is still lacking on the large scale. Yet, major components of synapses (such as synaptic boutons and spines) are undergoing continuous turnover and are actively maintained during adult life (e.g., [19]–[20]), which raises the possibility that information on synaptic connectivity may also be manifested in adult gene expression. This, coupled with the success of the earlier studies in the worm in predicting connectivity from adult gene expression [11]–[13], has motivated us to explore this possibility in depth here. The Allen atlas also provides a mapping between image regions and brain structures. By matching the brain structures of the rat connectivity map and the brain structures of the mouse brain we are able to construct a combined gene expression/connectivity atlas of the rodent brain (Materials and Methods). Using the combined atlas we find that gene expression levels in different brain regions contain considerable predictive information on their connectivity (interestingly, more than the level found in previous studies in the worm) and identify the genes and functional annotations whose expression is most predictive. Obviously some errors may be introduced in this mapping due to inter-species variations in connectivity and expression levels that may hinder the statistical significance of our results. Hence, importantly, the results presented here are likely to be a lower bound on the actual magnitude of the relationship between gene expression and regional brain connectivity. In parallel to our study, another group demonstrated evidence for a correlation between gene expression and connectivity in the rodent brain by using similar sources for gene expression of the mouse brain and rat connectivity maps [21].

## Results

The combined expression/connectivity atlas of the rodent brain contains 176 brain regions. Each is associated here with three signatures. The first signature is a gene expression vector of size 20,936 obtained from processing the Allen Brain Atlas. The other two signatures specify brain region connectivity: one encodes the outgoing connections from each region (Efferent connectivity), and the other encodes the incoming connections to each region (Afferent connectivity). Connectivity is obtained from the BAMS atlas [18] using the nomenclature of [22], assuming that connections that are not reported do not exist [23].

Similarly to [11] we study the connectivity information contained in gene expression by considering both prediction accuracy and the expression/connectivity correlation. Prediction accuracy measures the extent to which connectivity is predicted given the gene expression data. It is estimated for each region separately via a standard cross validation procedure. The correlation between gene expression and connectivity is a global index that measures how similar are the distances between regions in connectivity terms to their distances in expression terms, for all regions at once. On top of predictability and correlation, we also bring further support to our results by examining the enrichment of connectivity-related predicted genes in various disorders that are believed to be related to alterations in brain connectivity.

**Figure 1:**
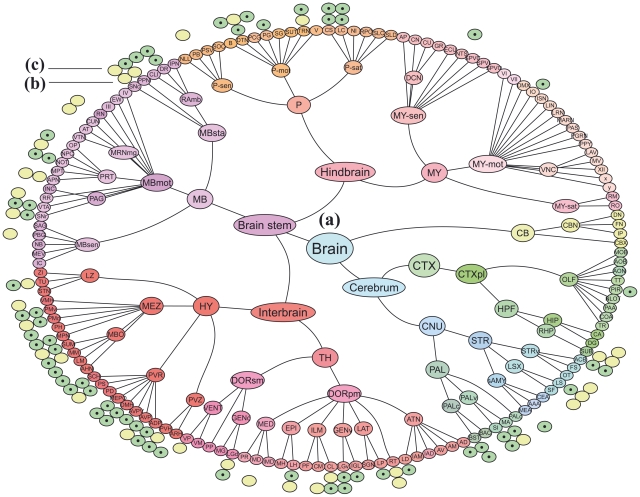
Brain regions for which prediction ability is significantly above chance. (a) The hierarchy of the brain regions in the Allen Brain Atlas is shown in the inner circle as circles with abbreviates. Colors are used to distinguish between subtrees. (b) Regions in the rodent brain for which prediction was significant at the *p* = 0.05 level for outgoing connectivity are marked green in the circle next to the outermost one. Those with insignificant prediction results are marked yellow. Note that only regions with at least 5 outgoing connections are marked by either color. (c) Similarly, for incoming connectivity in the outermost circle.

Connectivity prediction ability was studied using a linear SVM classifier (see Materials and Methods). We first obtain results for outgoing connections: In order to examine each region only once, we consider those 146 regions that do not contain other regions, i.e., regions that are leaves of the regional hierarchy of ABA (Figure 1(a)). Additionally, all regions that have less than 5 outgoing connections are discarded, resulting in a set of 44 regions A_1_,…,A_44_. We then fix a region A_i_ and consider the expression signatures of all other leaf regions B_1_,…, B_146_. At each of the 5 cross-validation iterations, we train a classifier using 4/5 of the regions and obtain a mapping between gene expression of the target region B_j_ and the existence of an outgoing connection from A_i_ to B_j_. The learned map is then applied to the remaining 1/5 regions in order to obtain predictions on the test data, unseen during training. These 5 iterations produce predictions to all regions B_1_,…,B_146_, and the overall prediction performance is quantified using the standard Area Under Curve (AUC) measure. A *p*-value is assigned to each region by performing a standard permutation test (see Materials and Methods). An analogous procedure was applied for predicting incoming connections.

The resulting prediction ability for outgoing connectivity is significant (p<0.05) for 32 out of the 44 regions (73%). The average AUC was 0.74 over all regions, and 0.79 for the significant regions. Significant prediction ability was observed also for the incoming connections. There are 57 regions that are not contained in other regions and which have at least 5 incoming connections. Out of these regions 32 (56%) have statistically significant (p<0.05) prediction accuracy. The average AUC is 0.73 for all the 57 regions and 0.83 for the 32 significant ones. The results for the prediction experiments (combining incoming and outgoing) are provided in Table S1, and the significant regions are portrayed in Figure 1(b,c). The outgoing and the incoming experiments share 35 brain regions that have at least 5 outgoing and 5 incoming connections, out of which 15 are successfully predicted in both incoming and outgoing sets.

In several regions of the hierarchy, the BAMS atlas is more detailed than the Allen Brain Atlas, therefore there are known BAMS connections that exist between substructures of the given leafs of the Allen Brain Atlas. In our study, such connections are eliminated since they arise from localized substructures that might have specific gene expression profiles, not necessarily matching that of the larger structures. This conservative approach is in line with the incompleteness of BAMS [24], i.e., the conservative connectivity map is geared to allow for more missing links rather than erroneously including spurious ones. However, for completeness, we also report the results obtained when taking a more liberal approach, which propagates links between BAMS substructures up to regions that have ABA analogs, are also presented in Table S1. This ‘liberal’ connectivity matrix contains well studied links that do not appear in the conservative connectivity map, such as the projection from the dentate gyrus to Ammon's horn. In this experiment too, there are many regions for which the connectivity prediction is significantly above chance −49% of the efferent regions and 58% of the afferent regions show significant predictability. While this is somewhat lower than the results obtained using the conservative connectivity matrix (73% and 56%), this drop in performance is expected due to the addition of regions with only few known connections, and the specificity of the connections to and from sub-regions that go beyond the resolution of the maps.

Several other alternative choices were also made in order to demonstrate the robustness of the experimental design and results, and are also depicted in Table S1. When choosing a threshold of 10 connections instead of 5, the average AUC obtained is similar; When replacing the SVM algorithm with the ensemble algorithm gentleBoost [25], results remain similar or slightly improve. Interestingly, when using the Nearest Neighbor algorithm as the classifier, the results somewhat deteriorate, suggesting that the connectivity predicting patterns are not metrically related in a trivial manner. To provide further support to the validity of the prediction method in the face of missing connectivity data (as BAMS is probably not comprehensive [24]), we also run simulations on synthetic connectivity graphs where one can carefully control the level of missing information (Materials and Methods). The results show that it is possible to have significantly correct predictions even if a large majority of the connections are missing.

Supplementary Table S2 shows predictions for individual connections that were obtained by aggregating the results over individual brain regions. Shown are both connections which are known to exist (230 outgoing and 207 incoming) and newly predicted connections that currently have not been reported in the literature (416 outgoing and 390 incoming), obtained with the natural SVM detection threshold at zero.

**Figure 2:**
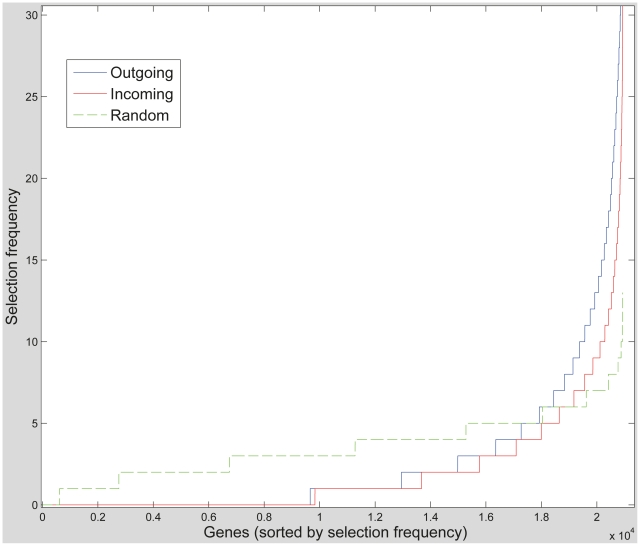
Frequency histogram of gene selection across regions. The number of times each of the 20,936 genes was selected as connectivity-predictive by the per-region zero-norm experiments. Graphs are shown for the Outgoing connections experiment, for the Incoming one, and compared to the frequency obtained with a random shuffle.

Using the connectivity prediction paradigm described above we employ a zero-norm SVM feature selection procedure (see Materials and Methods) to select the genes whose expression levels are most predictive of connectivity. For each region, the top 500 genes (out of 20,936) are selected, and a list of the 500 most frequently selected genes over all regions is formed, one for predicting the outgoing and one for predicting the incoming connections (Materials and Methods). As can be seen in Figure 2, many genes are selected repeatedly over the different regions in each of the outgoing and the incoming experiments.

**Figure 3:**
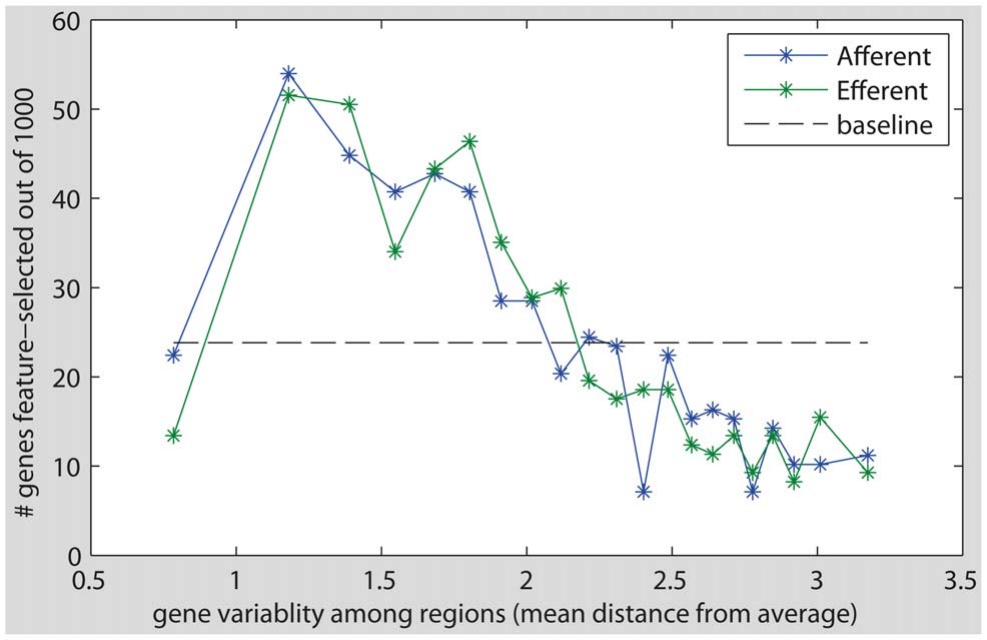
The correlation between variability in gene expression and predictability of connectivity. All genes were ranked by their region to region variability and put into equally sized bins. The intersection of each bin with the list of 500 efferent connectivity genes and the list of 500 afferent connectivity genes is shown (highest variance bin on the right, the x-axis depicts the amount of variability). As can be seen, the genes with the highest variability are not excessively frequent within the lists of most informative connectivity genes.

Remarkably, 241 genes (out of the 500 most selected) are shared by both the outgoing and the incoming lists (the expected number of shared genes according to the hypergeometric distribution is approximately 12, p<1e-5). The lists of genes selected are reported in Supplementary Table S3. Thus, in parallel to our finding that the connectivity of many brain regions is predictable on both the outgoing and incoming side, we also find that many genes are informative of both the incoming and outgoing connectivity. Since the outgoing predictions are based on the gene expression vectors of the target regions, and the incoming predictions are based on those of the source regions, the two sets of experiments use two halves of the data and the intersection of the two gene lists is not a statistical necessity. As a control test, we check whether those genes that show the highest region-to-regions variability are those that get selected as predictive. If this were the case, one could attribute their selection to the increased variability and not to their ability to predict connectivity. To this end, all genes were ranked according to their region-to-regions variability, measured as the mean distance from the average expression value, and put in equally sized bins. Then, the intersection of each bin with the two lists of the most informative genes was computed. As is evident from Figure 3 the selected connectivity-predicting genes are not necessarily those genes with the highest region-to-region variability and the two sets are inherently different. Apparently, a large amount of variability points to the influence of other factors that are not related to connectivity.

Having such lists gives as an opportunity to estimate the level of involvement of neural connectivity alterations in different brain disorders. To this end, we assembled from the literature lists of the top 100 genes that have been associated with each disorder examined, and quantified the number of (both efferent and afferent) connectivity related genes in each such list – the higher this number is, the more likely it is that connectivity alterations may play a role in the pathogenesis of the said disorder (Materials and Methods). Ranked by this measure (supp Table S4), the disorders we examined are (from the most associated to the least associated) Autism, attention deficit disorder, Schizophrenia, anxiety disorder, major depression, Parkinson's disease, bipolar disorder, Alzheimer's disease, obesity, glioma, and cardiovascular diseases. This ranking order fits fairly well with the prominent role ascribed to neuronal connectivity alterations in schizophrenia and autism. To obtain a rough estimate of the role of neuronal connectivity in these disorders as perceived in the literature, we recorded the number of web documents reported by the Google search engine that contained both the name of the disorder and the term “neuronal connectivity” and compared the latter to the connectivity-involvement measure we computed above. The web frequency count, as collected between March 28 and March 30, 2010 (supp Table S4), shows that the disorders examined can be divided to three main groups - high (schizophrenia and autism), low (obesity, glioma and cardiovascular) and medium level (the remaining ones). Quite remarkably, the high-frequency group has the highest mean of predicted connectivity related genes (15), followed by the medium level group (11.8) and then the low level one (3). These differences are statistically significant. Notably, one disorder originally belonging to the medium-level group (attention deficit disorder) has a similar number of connectivity-related genes as those in the high level group, possibly suggesting a potential role of connectivity alterations in its pathogenesis. A recent comprehensive meta-analysis of genes associated with Schizophrenia [21], listing 75 Schizophrenia related genes, has provided us an opportunity to examine our pertaining predictions in light of this gene association data. A random intersection of 500 genes would include less than 1.8 genes on average. The list of incoming connectivity genes intersects this list by 7 genes (p<0.002), and the outgoing lists intersects it by 4 genes (p = 0.1).

To estimate the global correlation (i.e., across all regions) between gene expression and connectivity we represent each of these two information sources as a square matrix that depicts the correlation in either gene expression or the connectivity profiles between every two regions (see Materials and Methods). Three 146×146 matrices are hence obtained: one based on similarity in gene expression and two for the similarity in incoming and outgoing connectivity profiles. Following previous work [11], [26], we compute the Pearson correlation between the lower triangular part of the matrices to evaluate correlation between data sources. The correlation between gene expression and outgoing connectivity is 0.26 (p<1e-7, empirical p<1e-4) and the one to outgoing connectivity is 0.23 (p<1e-6, empirical p<1e-4), showing again that there is a robust and significant relation between gene expression and regional brain connectivity.

We then employ such a correlation test to evaluate the connectivity information content of four different sets of genes of interest (Materials and Methods): an axon guidance list based on [27], a compilation of presynaptic genes [28], the list of predictive genes identified in *C. elegans* [11], and the list of genes that were found to bear an embryologic imprint [29]. The first two lists represent known gene sets that given their axonal/synaptic function are potentially, likely to be involved in determining and maintaining brain connectivity. The Third set has been previously found to be predictive in the worm. The last set might be correlated with connectivity since developmental relationships are sometimes mirrored in connectivity [30]. For each of these four sets we compute the 146×146 expression similarity matrix and examine its correlation to the original connectivity matrix obtained between the 146 different leaf regions. The results are presented in Table 1. Quite remarkably, only the genes known to be associated with axon guidance from the literature are significantly correlated with the brain regional connectivity and a significant correlation is absent for the three other groups. It is intriguing to find such an association between axon guidance and connectivity-related genes, even when looking at adult expression data. In addition to the four sets of genes, Table 1 presents the p-values of the connectivity correlation test applied to the lists of genes that were collected for each of the medical conditions mentioned above. These results are similar to the expected ranking, with various brain disorder genes showing an inter-region distribution that is significantly correlated with brain connectivity.

**Table 1:** Connectivity-information content in several pertaining groups of genes from the literature.

Functional annotation	Correlation test p-value	
	Efferent	*Afferent*
Axon guidence	0.05	0.13
Presynaptic	0.11	0.14
*C elegans*	0.63	0.31
Embryonic	0.32	0.71
Schizophrenia	<0.001	0.004
Autism	0.01	0.01
Bipolar	<0.001	<0.001
Alzheimer's disease	0.63	0.18
Major depression	0.01	0.02
Parkinson's disease	0.03	0.11
Attention deficit	0.11	0.52
Anxiety disorder	0.16	0.05
Obesity	0.22	0.19
Glioma	0.22	0.24
Cardiovascular	0.51	0.50

*p*-values are obtained using the correlation test (materials and methods).

To further study which gene annotation groups are informative with respect to connectivity, we also applied the correlation test to individual functional annotation groups. For each of 1,616 annotation groups in DAVID [31] that were at least partly expressed in the 20,936 genes at hand, we compute its 146×146 expression regional expression similarity matrix and examine its correlation to the original connectivity matrix. The results are summarized in Table 2 for the outgoing connectivity and Table 3 for the incoming connectivity, and are given in full in Table S5. Reassuringly, the top listed functional annotation groups are generally mostly related to neurogenesis, cell-cell signaling, synaptic activity and axonogenesis (both tables), and to neurotransmitter binding and receptor activity on the incoming side. There were 276 outgoing groups with p-value smaller than 0.05, and 200 incoming groups and the two lists share 156 annotation groups (18 expected by random).

**Table 2:** The gene annotation groups which were found to be most informative (p<0.001) with outgoing connectivity using the correlation test.

Functional annotation	Correlation	*p*-value
Axon	0.27	<0.001
Ionic channel	0.27	<0.001
Transmission of nerve impulse	0.26	<0.001
Axonogenesis	0.26	<0.001
Calcium transport	0.26	<0.001
Synaptic transmission	0.26	<0.001
Voltage-gated cation channel activity	0.26	<0.001
Cation channel activity	0.26	<0.001
Gated channel activity	0.26	<0.001
Ion channel activity	0.26	<0.001
Metal ion transmembrane transporter activity	0.26	<0.001
Substrate specific channel activity	0.26	<0.001
Channel activity	0.26	<0.001
Passive transmembrane transporter activity	0.26	<0.001
Neurite development	0.26	<0.001
Di-, tri-valent inorganic cation homeostasis	0.25	<0.001
Cellular morphogenesis during differentiation	0.25	<0.001
Neuron development	0.25	<0.001
Cellular di-, tri-valent inorganic cation homeostasis	0.25	<0.001
Blood circulation	0.25	<0.001
Neurogenesis	0.25	<0.001
Cell-cell signaling	0.25	<0.001
Neuron migration	0.25	<0.001
Homeostatic process	0.25	<0.001
Glycoprotein	0.25	<0.001

**Table 3:** The gene annotation groups which were found to be most informative (p≤0.001) with incoming connectivity using the correlation test.

Functional annotation	Correlation	*p*-value
Neurogenesis	0.25	<0.001
Ionic channel	0.25	<0.001
Calcium transport	0.25	<0.001
Neurotransmitter receptor activity	0.24	<0.001
Neurotransmitter binding	0.24	<0.001
Neuron migration	0.24	<0.001
Glycoprotein	0.24	<0.001
Blood circulation	0.24	<0.001
Axonogenesis	0.25	0.001
Gated channel activity	0.25	0.001
Substrate specific channel activity	0.24	0.001
Neuron projection	0.24	0.001
Neurite morphogenesis	0.24	0.001
Synapse	0.24	0.001
Metal ion transport	0.24	0.001
Chloride	0.24	0.001
Muscle contraction	0.24	0.001
Chloride channel	0.23	0.001
Gliogenesis	0.23	0.001
Neuromuscular process	0.22	0.001

Finally, it is interesting to compare the association we found between expression and connectivity of brain regions in rodents to the linkage previously found for single neurons in nematodes. To this end, we reanalyzed the data used in [11] using the global correlation test and created a list of functional annotation groups that are most correlated with connectivity in *C. elegans* (Table S6). A Pearson correlation test reveals that the list of *p*-values obtained for each functional annotation group in the worm is significantly correlated with the similar list obtained for rodents. For outgoing (incoming) connectivity, the correlation value is of 0.24, *p*-value 1e-5 (0.27, *p*-value 1e-6). Hence, there is a certain similarity in the functional gene groups that are associated with neural/brain connectivity across fairly distant phyla and across neuroanatomical scales.

## Discussion

Our work follows a direction set forth by previous work done for single neurons in *C. elegans* [11]–[13]. Despite obvious differences in the brain complexity, connectivity type, and the amount and quality of the data, it is interesting to compare the prediction performance obtained here to that of its preceding *C. elegans* investigation. In the previous study of [11], the mean Area Under the ROC curve (AUC) for the prediction experiments is only about 0.6 for both incoming and outgoing connectivity. In our results, the average AUC is markedly higher (0.73 and 0.74). For all 289 genes used in [11], the correlation between connectivity and expression in the worm was 0.176 for outgoing connectivity, and 0.075 for incoming connectivity. Looking at all of the 20 thousands plus genes used in this work at once, the equivalent correlations are 0.26 and 0.23. Moreover, there is considerable variance in the predictability in different regions and some regions achieve quite high predictive values (0.83 and 0.79 mean AUC values over the significant regions, with maximal AUC values reaching 0.99).

Our results are further supported by the recent parallel contribution of French and Pavlidis [21], in which a similar correlation test yields a score of 0.22 and 0.26 for incoming and outgoing connectivity respectively. The work of [21] is focused on the correlation assay and the authors state that they were unable to perform convincing predictive experiments. Here, in difference, we show that there is a considerable predictive signal. In fact, the prediction capability is considerably stronger than that found in the worm, and many of the brain regions present a marked and highly significant level of predictability. This prediction ability is further used here to select the lists of connectivity-related genes. A predictive test is, in our minds, a more solid foundation for gene selection than a correlation test. This is because a combination of even uninformative features can produce a correlation map that is similar to a given input map, while the separation between train and test data in the prediction experiments is much less prone to this pitfall. The lists of selected connectivity-related genes we obtain are verified here by comparing them to various lists obtained from the literature, again, going beyond the results presented in [21].

Regions of high predictability do not seem to be clustered in specific parts of the hierarchy. While smaller nuclei with many connections and therefore more available data seem somewhat easier to predict, a comparison between a structure's volume and the predictability of its connectivity map shows that regions of all sizes depict good predictability (Supplementary Figure S2). This might suggest that all regions are potentially of high predictability; however, the quality of the data currently available limits our ability to uncover their true predictability. The correlation between spatial proximity and connectivity is 0.11 and 0.10 for outgoing and incoming connectivity (compared to 0.26 and 0.23). Thus, while in the brain nearby regions are more likely to be connected, this association is significantly lower than the association between gene expression and connectivity.

To build the combined rodent brain atlas that contains both expression and connectivity, we rely on available resources that are not fully compatible or complete. Some of the connectivity that is currently absent in the rat atlas may actually exist in the rodent brain. The assumption of conservation of connectivity and expression between mouse and rat, underlying the construction of a combined atlas of a common rodent ancestor, probably holds only partly. Furthermore, the gene expression data was not measured during brain development, as would ideally have been more befitting. Yet, as both connectivity and expression are associated with common factors such as functionality, it is perhaps not surprising that considerable pertaining information can be delineated in adult expression patterns of neurons. As evident, the latter permit a considerable level of connectivity prediction, exhibit significant correlations with the connectivity data, and show a marked overlap between genes that are discriminative for incoming and outgoing connectivity. Finally, strictly speaking, we identify an association and not a causal relation from genes to connectivity. Although this causal direction is expected based on current consensus, it is certainly possible that connectivity in turn affects gene expression – one possible route for such effects may be indeed via activity-dependent mechanisms that shape synaptic formation and maintenance, mentioned earlier [8]–[9].

Despite the above limitations to the quality of the data, we were able to uncover a fairly marked association between gene expression and connectivity. Thus, we are able to make a significant advancement toward the long term goal of inferring the connectome from the genome [32]. Naturally, had our data been richer, for example, alleviating the need to rely on conservation across species, even better results could be expected. However, especially given these limitations, the magnitude of the association found here is truly remarkable, and the large-scale analysis approach presented here will undoubtedly show its continuing value in future studies as more refined data accumulates. This type of analysis is valid for both single neuron connectivity and connectivity between brain regions, and it is likely to be valid for intermediate, mesoscopic scales [24], [33]. In the nearby future, such efforts can be applied to link between newly established connectivity maps in humans (e.g. [34]) with accumulating regional gene expression data in the human brain. Moreover, once the genetic atlas of the developing brain [16] is processed to register gene maps, a distinction can be drawn between genes that are associated in maintaining connectivity and genes that are dominant during the initial formation of brain connectivity.

With the future advent of better and more accurate data we might be able to perform the analysis presented here focusing solely on the gene expression of neuronal cells while disregarding other cell types. To gain preliminary experimental insight into the role played by cell type in determining the link between expression and connectivity, we have examined the human data available from two recent papers. The first paper [35] has microarray data collected from the brains of AD patients and controls. In the second paper [36], care was taken such that the gene expression data was collected from neurons only. Therefore, for a first approximation, we have samples that are glia + neurons and samples that are only neurons. By comparing the two sets of samples we can identify genes that are over-expressed in glia and not over-expressed in neuron samples (Note that the situation is not symmetric and the opposite list cannot be extracted without further assumptions). Working with the mouse homologs of the identified human genes, we find that those genes that tend to be over expressed in glia are less informative than a typical group of the same size. The p-value of this finding is borderline though – 0.02 for efferent correlation test and 0.17 for the afferent correlation test. Future studies analyzing neuronal vs glial expression data comparatively are hence needed to shed further light on this intriguing question.

## Materials and Methods

### Data and preprocessing

Our study has been made possible thanks to the innovative open approach of the Allen Brain project [16]. Gene expression data was obtained from the Allen Mouse Brain Atlas (ABA) dataset [15] for gene expression in the adult mouse brain composed of 20,936 genes (http://mouse.brain-map.org/). For each gene a 200 micron 3d volume of gene expression in the mouse brain is available (a vector of length ∼150 k). Some genes have several scans. Scans are available in one of two planes: Coronal and Sagittal. We compiled a dataset of voxel gene expressions based on sagittal scans. When numerous scans exist for a single gene a mean is taken (maximum was also tried – resulting in only minute, negligible differences in results reported).

For linking voxels to brain structures we use the structural annotation available at ABA (http://mouse.brain-map.org/pdf/Allen_Reference_Atlases.pdf). It defines a nomenclature of 209 brain structures organized in a hierarchy. The gene expression for each brain structure is computed as the average of all voxels contained within that region. Once more, experiments were also performed by taking the maximal value instead of the mean with little, negligible influence on the connectivity prediction ability and on the results reported.

One should note that during the preparation of this work partial results on the developing mouse brain have been uploaded to the ABA website. These results are not complete enough to enable us to run our experiments on a developing brain. For example, there is no mapping currently available between voxels and brain structures.

Rat connectivity information is obtained from [18]. To match rat connectivity to mouse gene expression we link the rat nomenclature of [22] and the ABA mouse nomenclature, by creating a mapping between identical terms. The mapping is given in Table S7.

It sometimes occurs that a region is identified in the mouse nomenclatures and at least one of the children of this region is not identified. Even in such cases, we do not perform the analysis on the non-leaf regions. This policy simplifies the framework and minimizes borderline cases, for example, when some of the leaves are identified and some are not.

### Prediction assay

We use a Linear Support Vector Machine (SVM) [37] classification with a fixed parameter of C = 1 for prediction. The learned binary labels correspond to the existence or non-existence of a connection between regions. Regions with less than 5 positive examples (i.e. connections) are discarded. For each region separately, a balanced 5-fold cross-validation is performed on this data with 80% training and 20% testing. Since each connection (existing or not) is tested exactly once, the cross validation procedure produces a connectivity prediction value for each possible connection. We consider the real value which is the signed distance from the learned classifier's separating hyperplane, and use it to compute the Area Under Curve (AUC) statistics. To eliminate dependence on the random split used, each such cross-validation experiment is repeated 20 times, and the mean AUC is recorded. In order to evaluate statistical significance, the entire experiment is repeated 1,000 times while permuting the labels.

To demonstrate the validity of the prediction assay in the face of missing connectivity data we perform the following synthetic data experiment: A random network was created of a similar cardinality as the BAMS network used in our experiments, such that the degrees of the nodes are five times higher than those of the BAMS network (varying between nodes, similarly to BAMS). Synthetic random vectors of “gene expression” were created in such a way that nodes that are connected to a specific node have for a subset of the genes a somewhat similar pattern, randomly varied around a certain central pattern, i.e., tend to have some genes overexpressed and some genes underexpressed in a similar manner. Then, we run the same protocol as in our prediction assay and measure success by computing the mean AUC obtained from all regions (the equivalent success in the real data experiments is 0.73). This experiment is then repeated when some of the initially given positive connections are held out and marked as ‘non existing’ (i.e., incorporating missing data in a controlled manner).

The results of the simulations for specific missing data values, averaged over many runs are presented below in Supplementary Figure S1 . As can be seen, even for such challenging simulations where the prediction for the full dataset is at 80%, the results degrade nicely with the number of missing connections. In these noisy conditions the results vs the simulated atlas remain well above chance even when only 15% of the connections are retained (i.e., ‘known’, blue-line). Moreover, the classifiers learned with the missing data are useful for predicting the complete (no missing data) simulated connections (red-line).

### Correlation assay

To examine the correlation between a genetic pattern and a connectivity pattern across all brain structures under investigation, we used an assay similar to the one used by Toledo–Rodriguez et al [26]. This assay was also used in [11]. Given a set of *N* = 146 structures, we constructed two *N×N* similarity matrices, *S_1_* and *S_2_*, where *S_1_* (*S_2_*) represents the pairwise similarity between the expression data (connectivity) of every two brain structures. Pearson correlation is used as a measure of those pairwise similarities for both gene expression and connectivity, both between the vectors of gene expression, and the connectivity vectors. The (N * N/2 – N) entries forming the lower triangle of *S_1_* (*S_2_*) are concatenated to form a covariation vector *v_1_* (*v_2_*). The Pearson correlation between the two covariation vectors *v_1_* and *v_2_* describes the extent to which similarities in gene expression imply similarities in connectivity and vice-versa. The statistical significance of the resulting correlation is computed using an empiric null hypothesis constructed from repeating the procedure with shuffling. On each repetition the gene expression signatures were shuffled amongst all regions, thus disassociating a region and its gene expression. The *p*-values are calculated by repeating the shuffling 1,000 times and computing the probability to achieve a score equal or higher than the score of the non-shuffled data.

### Feature (gene) selection

Similarly to the prediction assay, for each brain region we take connected regions gene expression as positive examples and non-connected regions as negative examples. This is done once for outgoing connections, and once for incoming connections, where the two experiments are performed independently. At each time, feature (gene) selection was performed using *zero norm SVM* algorithm [38]. Zero norm SVM works by iteratively training an SVM while reweighing the feature vectors until convergence. In order to select a fixed number of features, we have selected the 500 features with the highest weights provided by the zero-norm SVM procedure. This is repeated for each brain structure which has at least 5 connections, i.e., to 44 regions in the outgoing experiment and to 57 regions in the incoming experiment.

To obtain two global lists of selected genes that are informative to either outgoing connectivity or incoming connectivity, the individual lists obtained for each region are combined. This is done by counting for each gene the number of times it was selected across the brain structures in each of the two experiments. The 500 genes that appeared most frequently in the individual outgoing experiments form the list of selected outgoing genes, and similarly for the incoming list.

To gain more insight into the nature of the selected genes, we have employed the DAVID functional annotation tools [31] to determine the most prominent annotations in the two lists formed above. The details of this experiment are provided in Supplementary Table S8.

To alleviate potential concerns about the influence of artifacts in the gene expression data on the prediction and gene selection process, we have compared the prevalence of artifacts in the data of selected genes to that of a disjoint sample of genes. 50 genes were sampled randomly from the groups 241 genes that are found to be predictive for both outgoing and incoming connectivity. Another group of 50 genes was sampled from the 1000 most brain active genes that do not appear in either list of predictive genes. For further control, genes that were not highly expressed in the brain were removed from the study since their images are expected to contain less data and therefore fewer artifacts.

The results show that for the sample of connectivity predictive genes, 58% of the slices contained local artifacts such as localized stains. The equivalent number for the background group is 57%. The ratio of global artifacts such as folds and scratches are also quite similar between the two groups: 11% and 17% respectively. Overall, we do not observe a tendency for more artifacts in the selected genes in comparison to the general population of brain-expressed genes. Supplementary table S9 contains the raw data of this analysis.

### Medical disorder gene-lists

The top 100 genes associated with each disorder were extracted from the HuGe database [39], and the size of the intersection of these lists and the two lists of connectivity genes extracted by the feature selection method above were computed. The expected size of a random intersection is 2.5 genes.

### Literature based gene-lists

There were 4 such lists. (1) Axon guidance genes were obtained from the gene families discussed in [27]: Netrin, Slit, Semaphorin, Ephrin, DCC, UNC5, Robo, Robo3, Neuropilin, Plexin and Eph. A total of 86 homologous members of these families were matched in the ABA gene set. (2) A group of 103 pre-synaptic gene homologs was obtained from a list of 107 genes appearing in [28]. (3) *C.elegans* genes were obtained from mouse homologies on the most highly ranked genes shown to be involved in neural connectivity in [11]. ABA homologies of 19 outgoing (31 incoming) were obtained from 30 outgoing (53 incoming) *C.elegans* genes. (4) The list of genes which are indicative of embryonic history taken from [29]. 83 such genes were identified within the ABA gene list out of 93 in the original list.

### Computing significance of the correlation obtained by a group of genes

In order to compute the significance of the correlation assay results obtained by a group of genes, such as the three literature based gene-lists or the 1,616 DAVID groups, we have compared the p-value obtained using the correlation assay with the p-values obtained for 1000 random groups of the same size. This procedure eliminates bias caused by the group size.

## Supporting Information

Figure S1 Area Under Curve (AUC) of synthetic data experiment as a function of the amount of missing data. The blue curve shows the AUC when using the degraded labels (those with missing values) to compute the ROC curve. The red curve shows the AUC obtained with full labels.
(TIF)

Figure S2 A plot showing a comparison of the volume (in cubic mm.) of each substructure (as obtained from the ABA) to the p-value in the prediction experiment.
(TIF)

Table S1 Prediction assay results.
(XLS)

Table S2 Prediction results are compared to the BAMS ground truth. For each potential connection (existing or not) that participates in the prediction experiment we report the BAMS data and the prediction result, which is obtained when the potential connection is part of the test data.
(XLS)

Table S3 Selected genes (500 outgoing, 500 Incoming, and 241 intersection).
(XLS)

Table S4 Linking between medical conditions and brain connectivity by document count and by number of genes that appear both in the relevant association study and in the genes selected as informative for connectivity.
(XLS)

Table S5 Functional annotation groups that were found to be informative by the correlation test for the rodent data.
(XLS)

Table S6 Correlation test applied to the functional annotation groups arising from 142 genes for which there is information for both rodents and *C. elegans*.
(XLS)

Table S7 Mapping between rat and mouse brain region nomenclatures.
(XLS)

Table S8 DAVID Functional annotation analysis for the 500 selected genes.
(XLS)

Table S9 Prevalence of artifacts in the ABA gene expression images, comparing the selected genes (Table S3) with a background set of genes that are expressed in the brain.
(XLS)
