# Supplementary figures and images for: Gene Expression in the Rodent Brain is Associated with Its Regional Connectivity
Source: PLoS Comput Biol. 2011 May 5;7(5):e1002040. doi: 10.1371/journal.pcbi.1002040 (PMC3088660; doi:10.1371/journal.pcbi.1002040)

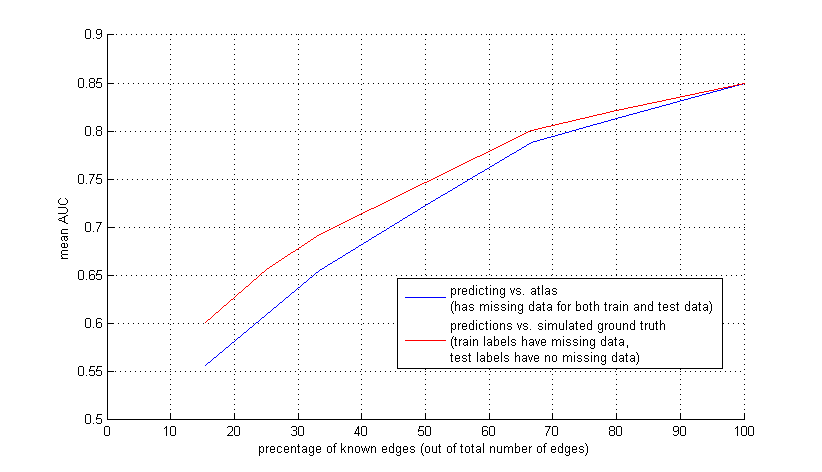

Supplement: Figure S1 — Area Under Curve (AUC) of synthetic data experiment as a function of the amount of missing data. The blue curve shows the AUC when using the degraded labels (those with missing values) to compute the ROC curve. The red curve shows the AUC obtained with full labels. (TIF) [file pcbi.1002040.s001.tif]

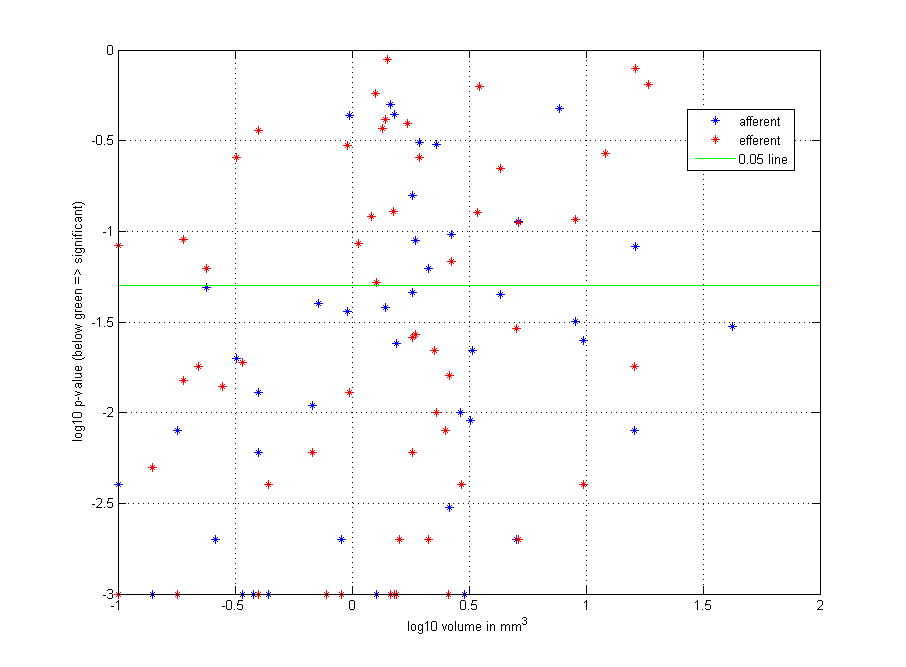

Supplement: Figure S2 — A plot showing a comparison of the volume (in cubic mm.) of each substructure (as obtained from the ABA) to the p-value in the prediction experiment. (TIF) [file pcbi.1002040.s002.tif]
